# Stimulating the nucleus accumbens in obesity: A positron emission tomography study after deep brain stimulation in a rodent model

**DOI:** 10.1371/journal.pone.0204740

**Published:** 2018-09-27

**Authors:** Marta Casquero-Veiga, David García-García, Javier Pascau, Manuel Desco, María Luisa Soto-Montenegro

**Affiliations:** 1 Instituto de Investigación Sanitaria Gregorio Marañón (IiSGM), Madrid, Spain; 2 Facultad de Ciencia y Tecnología, Universidad Isabel I, Burgos, Spain; 3 CIBER de Salud Mental (CIBERSAM), Madrid, Spain; 4 Departamento de Bioingeniería e Ingeniería Aeroespacial, Universidad Carlos III de Madrid, Leganés, Spain; 5 Centro Nacional de Investigaciones Cardiovasculares (CNIC), Madrid, Spain; Oslo Universitetssykehus, NORWAY

## Abstract

**Purpose:**

The nucleus accumbens (NAcc) has been suggested as a possible target for deep brain stimulation (DBS) in the treatment of obesity. Our hypothesis was that NAcc-DBS would modulate brain regions related to reward and food intake regulation, consequently reducing the food intake and, finally, the weight gain. Therefore, we examined changes in brain glucose metabolism, weight gain and food intake after NAcc-DBS in a rat model of obesity.

**Procedures:**

Electrodes were bilaterally implanted in 2 groups of obese Zucker rats targeting the NAcc. One group received stimulation one hour daily during 15 days, while the other remained as control. Weight and daily consumption of food and water were everyday registered the days of stimulation, and twice per week during the following month. Positron emission tomography (PET) studies with 2-deoxy-2-[^18^F]fluoro-D-glucose (FDG) were performed 1 day after the end of DBS. PET data was assessed by statistical parametric mapping (SPM12) software and region of interest (ROI) analyses.

**Results:**

NAcc-DBS lead to increased metabolism in the cingulate-retrosplenial-parietal association cortices, and decreased metabolism in the NAcc, thalamic and pretectal nuclei. Furthermore, ROIs analyses confirmed these results by showing a significant striatal and thalamic hypometabolism, and a cortical hypermetabolic region. However, NAcc-DBS did not induce a decrease in either weight gain or food intake.

**Conclusions:**

NAcc-DBS led to changes in the metabolism of regions associated with cognitive and reward systems, whose impairment has been described in obesity.

## Introduction

Obesity is defined as abnormal or excessive fat accumulation which may impair health [1]. The prevalence of obesity has reached epidemic levels, and its comorbid conditions cause at least 2.8 million deaths per year worldwide [2]. In addition, obesity is a risk factor for many other highly prevalent diseases [3].

Initial anti-obesity treatments focus on diet and exercise routines [4]. However, in refractory patients, specialists turn to pharmacological and surgical procedures, which can cause serious adverse effects and fail to control the disease in the long term [5,6]. Therefore, new therapeutic approaches must be found to reduce the prevalence of obesity. Among them, deep brain stimulation (DBS) seems promising for treatment-resistant obesity. This therapy involves sending electric stimuli from a pulse generator to brain nuclei via electrodes in order to modify impaired function. However, the mechanism of action of this approach remains unknown. In this sense, the traditional concept of an ablative effect of high-frequency (HF) DBS (100–160 Hz) [7,8] is now being replaced. In fact, there is growing interest in alternative proposals, such as the idea that HF-DBS could cause an ‘informational lesion’ [9] or disruption of the neural informational flow [10] in the target structure, which would produce therapeutic benefits. Therefore, inadequate signals from a specific nucleus could be isolated by stimulating a downstream target and hence correcting the malfunction of the neural circuit. In any case, DBS offers important benefits over ablative neurosurgery, such as reduced invasiveness, possible reversibility, and the possibility of *in vivo* adjustment of the stimulus applied [11]. Furthermore, several authors have shown stereotaxic surgery to be safe in clinical procedures [12–15], thus reinforcing its potential application to a broader range of diseases. In this sense, DBS has been validated as a palliative treatment in motor diseases [16] and obsessive-compulsive disorder [17], and its potential role has been investigated in other neuropsychiatric disorders [18].

The nucleus accumbens (NAcc) has received much attention as a key target structure of the reward system in the treatment of obesity [19–21]. Therefore, NAcc-DBS could modulate the reward processes related to food intake and lead to weight reduction [7,20,22]. Two case reports assessing bilateral NAcc-DBS in obese patients show significant weight loss [23,24]. Interestingly, the second report described a patient with pathological obesity due to craniopharyngioma surgery [24], thus highlighting the interaction between the homeostatic and reward mechanisms involved in feeding. The communication between these neural systems would be mediated by an interplay between the lateral hypothalamus (LH), ventral tegmental area (VTA), and NAcc, in which leptin would play a central role [20].

Neuroimaging offers a variety of powerful tools to study the regions involved in the pathophysiology of obesity, as well as those modulated by DBS. In particular, positron emission tomography (PET) with 2-deoxy-2-[^18^F]fluoro-D-glucose (FDG) is a suitable technique for characterizing functional neuronal networks in small animals, and has proven useful for elucidating the mechanism of action of DBS [25,26]. In fact, we previously showed that LH-DBS induced metabolic changes in brain regions related to the control of food intake and reduced weight gain in a leptin signal–deficient model of obesity (obese Zucker rat) [27].

**Fig 1:**
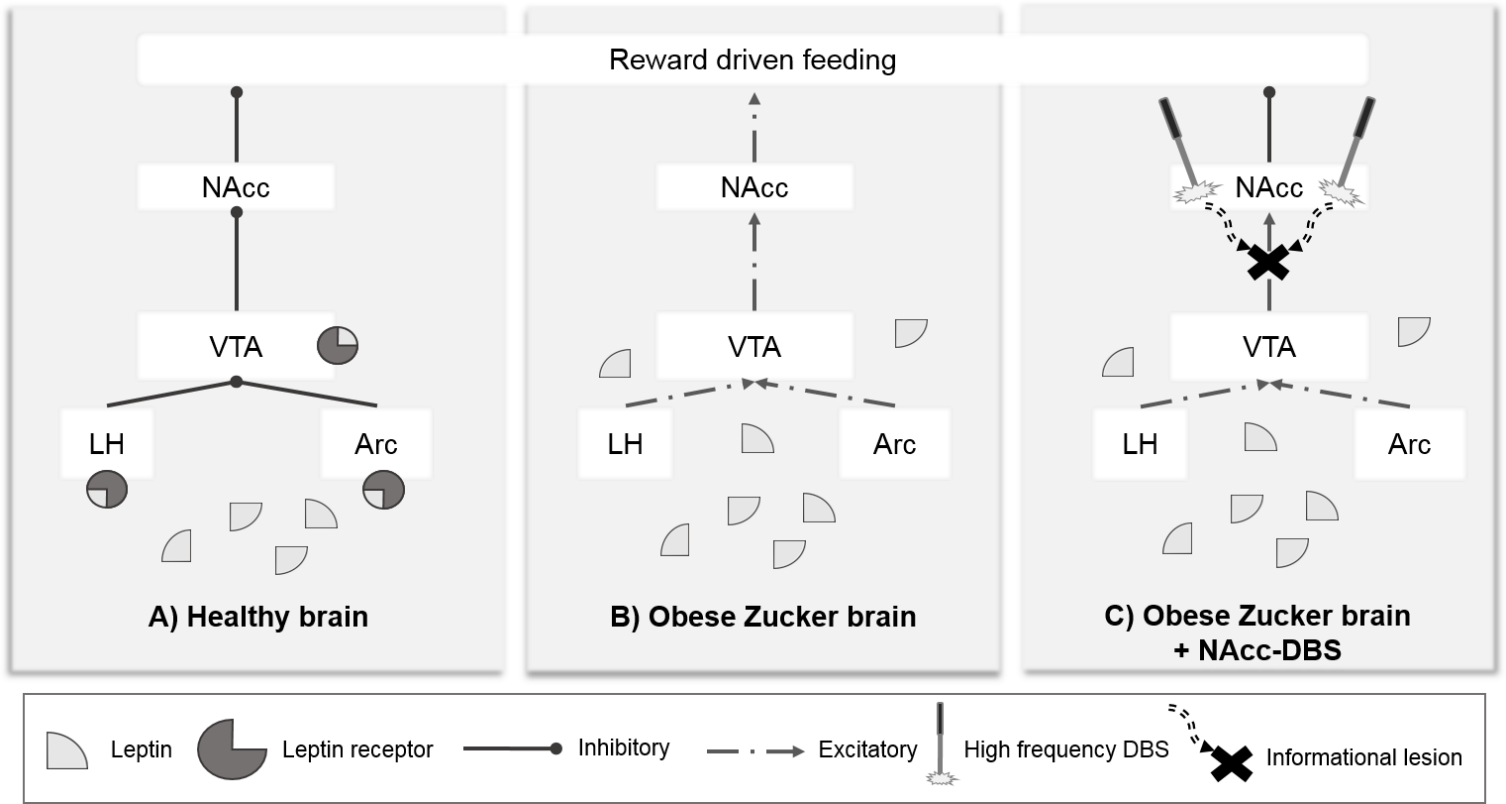
Study hypothesis. Schematic explanation of the hypothalamic-mesolimbic brain circuit state in the following: A) healthy brain; B) obese Zucker brain; and C) obese Zucker brain with NAcc-DBS, showing the disruptive mechanism of action theory [9,10]. Partially adapted from [20,28] [Arc: arcuate, LH: lateral hypothalamus, VTA: ventral tegmental area, NAcc: nucleus accumbens].

Given this background, and considering the hypothesis that DBS can block the impaired signaling sent by VTA and LH in the absence of the influence of leptin, we assessed the metabolic changes induced by NAcc-DBS in our previous animal model by applying an identical DBS protocol [20] (see Fig 1). As a result, the stimulation could reduce food intake and, hence, weight gain.

## Material and methods

### Animals

The obese Zucker rat was selected as an animal model of treatment resistant obesity, which is representative of the potential beneficiaries of this therapy. It is homozygous for a truncated form of the leptin receptor and hence has genetic resistance to this hormone. Leptin is released by adipose tissue in proportion to its extension and the amount of lipids ingested during meals. It targets the lateral LH, ventromedial hypothalamus and VTA [20,29], and acts as a signal to stop eating. Consequently, Zucker rats experience hyperphagia, hyperinsulinemia, and hyperlipidemia; which lead to spontaneous obesity [30].

**Fig 2:**
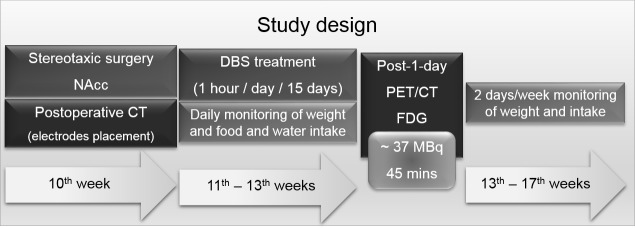
Study design. Design of the experimental procedures performed during the study in relation to the age of the animals.

In this work, fifteen adult male obese Zucker rats (fa/fa^-^, Charles Rivers Laboratories, Spain) (10-week old) were housed individually in a temperature- and humidity-controlled room on a 12 h dark/light cycle with food (standard laboratory chow) and water available *ad libitum*. Weight, food and water consumption were monitored daily during the 15 days of stimulation, and twice per week during the following month. Measurements were always collected at the same time of the day. Prior to the PET study, animals were deprived of food but allowed free access to water for 6–8 hours. The study design is shown in Fig 2.

All experimental animal procedures were conducted according to European Communities Council Directive 2010/63/EU and approved by the Ethics Committee for Animal Experimentation of Hospital Gregorio Marañón.

### Surgery

**Fig 3:**
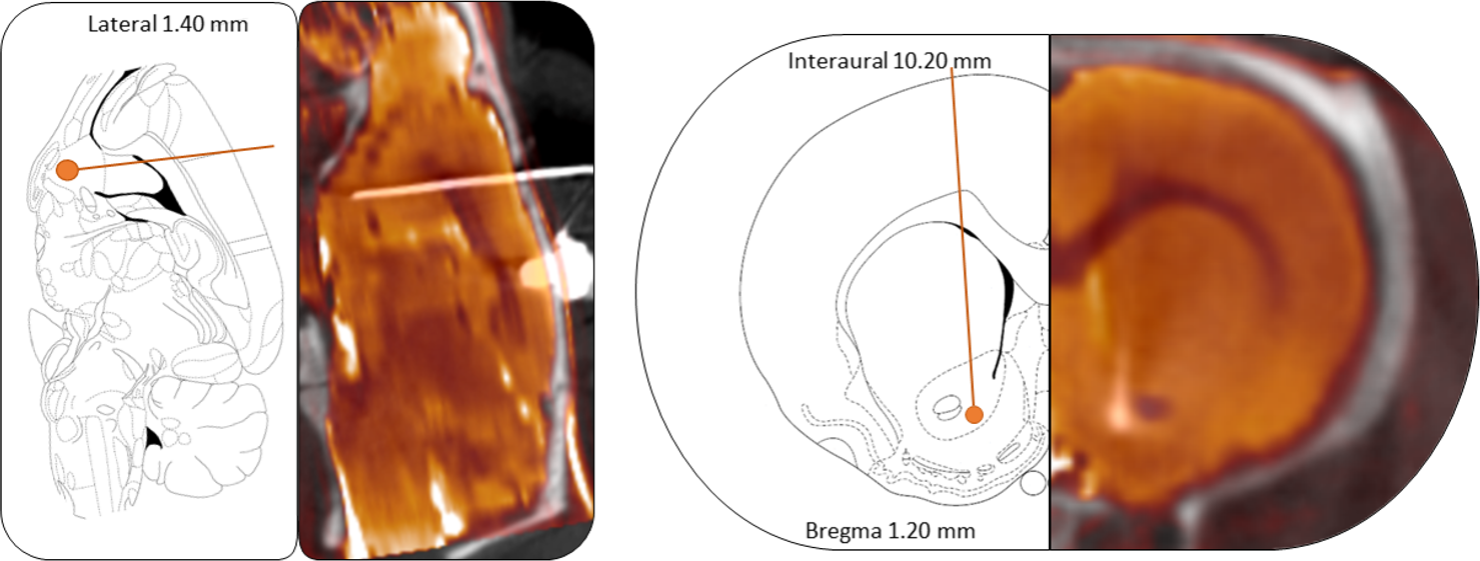
Electrodes placement verification. Representative sagittal (left) and axial (right) views of a CT scan registered to the MR template of an animal next to the correspondent slice from [31] to verify the correct electrode location in the NAcc.

Stereotaxic procedures were performed at 10 weeks-age under a mixture of ketamine/xylazine (100/10 mg/kg). Concentric bipolar platinum-iridium electrodes (MS303/8-AIU/Spc, Bilaney Consultants GmbH, Germany) were bilaterally implanted to target the NAcc core (+1.2 mm posterior and +1.5 mm lateral from bregma, -8.2 mm ventral from the dura) [31]. Electrodes were fixed to the skull bone with acrylic dental cement (Technovit, Heraeus-Kulzer, Germany) reinforced with four small stainless steel screws attached to the skull. Ceftriaxone (100 mg/kg IM) and buprenorphine (0.1 mg/kg IP) were administered during 5 days as postoperative care. The correct electrode location verification is shown in Fig 3.

Remarkably, although the electrodes were implanted in the NAcc core, the selected stimulation protocol is expected to directly affect a wider area, including also the NAcc shell [32]. Therefore, we will refer to the NAcc as the target of stimulation, without distinguishing between subregions.

### DBS protocol

DBS started 7 days after surgery to allow the animals sufficient recovery time. Animals were divided into 2 groups: NAcc-sham (*N =* 9) (surgery with electrodes implantation but no stimulation) and NAcc-DBS (*N =* 6) (surgery plus stimulation). As animals could freely move when receiving the stimulation into their cages, the stimulator wire was susceptible of snagging due to animals movements. Therefore, NAcc-sham animals were not plugged to the stimulator in “off position” in order to avoid losing any surgical implant.

DBS was performed with an isolated stimulator device (CS 120 8i, CIBERTEC S.A., Spain) set at a constant current of 150 μA (130 Hz) and a pulse width of 100 μs (biphasic stimulation mode). Stimulation was applied for 1 hour/day over 15 days. These settings were chosen based on previous preclinical and clinical studies [25,33,34].

### Imaging studies

PET studies were acquired one day after the DBS protocol finished with a small-animal PET/CT scanner (ARGUS PET/CT, SEDECAL, Spain), under anesthesia with isoflurane (3% induction, 1.5% maintenance in 100% O_2_). 2-deoxy-2-[^18^F]fluoro-D-glucose (FDG) (~37 MBq) was injected through the tail vein, and animals were scanned for 45 min. Images were reconstructed using a 2D-OSEM algorithm, Full Width at Half Maximum (FWHM) of 1.45 mm, with a voxel size of 0.3875 x 0.3875 x 0.775 mm^3^ and an energy window of 400–700 keV. Decay and dead-time corrections were applied.

We obtained two CT scans for each animal: at the end of the surgery to check the correct placement of the electrodes, and simultaneously with the PET studies. CT studies were acquired with the same scanner (340 mA, 40 kV, 360 projections, 8 shots, and 200 μm of resolution) and reconstructed using an FDK algorithm (isotropic voxel size of 0.121 mm) [35]. Only animals with a correct placement of the electrodes were included in the study.

An MRI scan of a single non-operated animal was acquired with a 7-Tesla Biospec 70/20 scanner (Bruker, Germany) for use as anatomical template in the statistical analyses. A T2 spin-echo sequence was acquired, with TE = 33 ms and TR = 3732 ms. The scan parameters were as follows: 34 slices measuring 0.8 mm in thickness; matrix size 256x256 pixels; and FOV of 3.5x3.5 cm^2^. The artifact caused by the surface coil was corrected.

### Data processing and statistical analysis

#### Intake and body weight

Daily food and water intake during the DBS period, as well as average food ingested every 5 days, were used to evaluate the real consumption, as daily consumption is a very noisy variable. Body weight results are expressed as the difference in weight (%) with respect to baseline. Changes in weight and intakes were evaluated with GraphPad Prism version 5.00 (GraphPad Software, USA), using a 2-way ANOVA to compare both groups. Moreover, we used linear regression to evaluate the progression of weight changes from baseline, comparing the obtained slope for each group by an ANCOVA analysis.

#### PET data

PET data followed a preprocessing registration protocol previously described[27]. Briefly, PET scans were co-registered to a random reference CT scan by an automatic method based on mutual information [36]. The MRI scan was also registered to the same spatial frame with the same method. Images were studied by voxel-by-voxel and region of interest (ROI) analyses. For the former methodology, PET registered images were normalized to global mean brain intensity in accordance to Shinohara *et al*. criteria [37], and smoothed using a Gaussian kernel of 0.96875 x 0.96875 x 1.9375 mm^3^ of FWHM. A whole brain (WB) mask was segmented from the registered MRI study and applied to all PET images in order to eliminate voxels outside the brain. Then, we performed a voxel-by-voxel analysis of data using SPM12 (http://www.fil.ion.ucl.ac.uk/spm/software/spm12/). Groups were compared using a two sample T-test, setting a significance threshold of p<0.005 uncorrected (voxel-level significance), but cluster-based corrected in order to avoid type II errors [38]. Moreover, only significant regions larger than 50 activated connected voxels were accepted aiming at reducing type I error.

ROI analysis was performed to discard global differences in brain metabolism in order to confirm the validity of WB as normalization region. Moreover, we studied other brain areas with the aim of ratifying the previously observed group differences in the voxel-by-voxel procedure. Thus, masks from WB, NAcc, caudate-putamen (CPu), thalamus (Th) and cortex (Cx) were segmented from the registered MRI. WB data was evaluated by means of standardized uptake values (SUV); while the remaining ROIs data were normalized to the mean intensity of the WB mask. Analyses were performed by a two sample T-test (p<0.05) in GraphPad Prism version 5.00.

## Results

### *In vivo* study of the DBS effect

**Fig 4:**
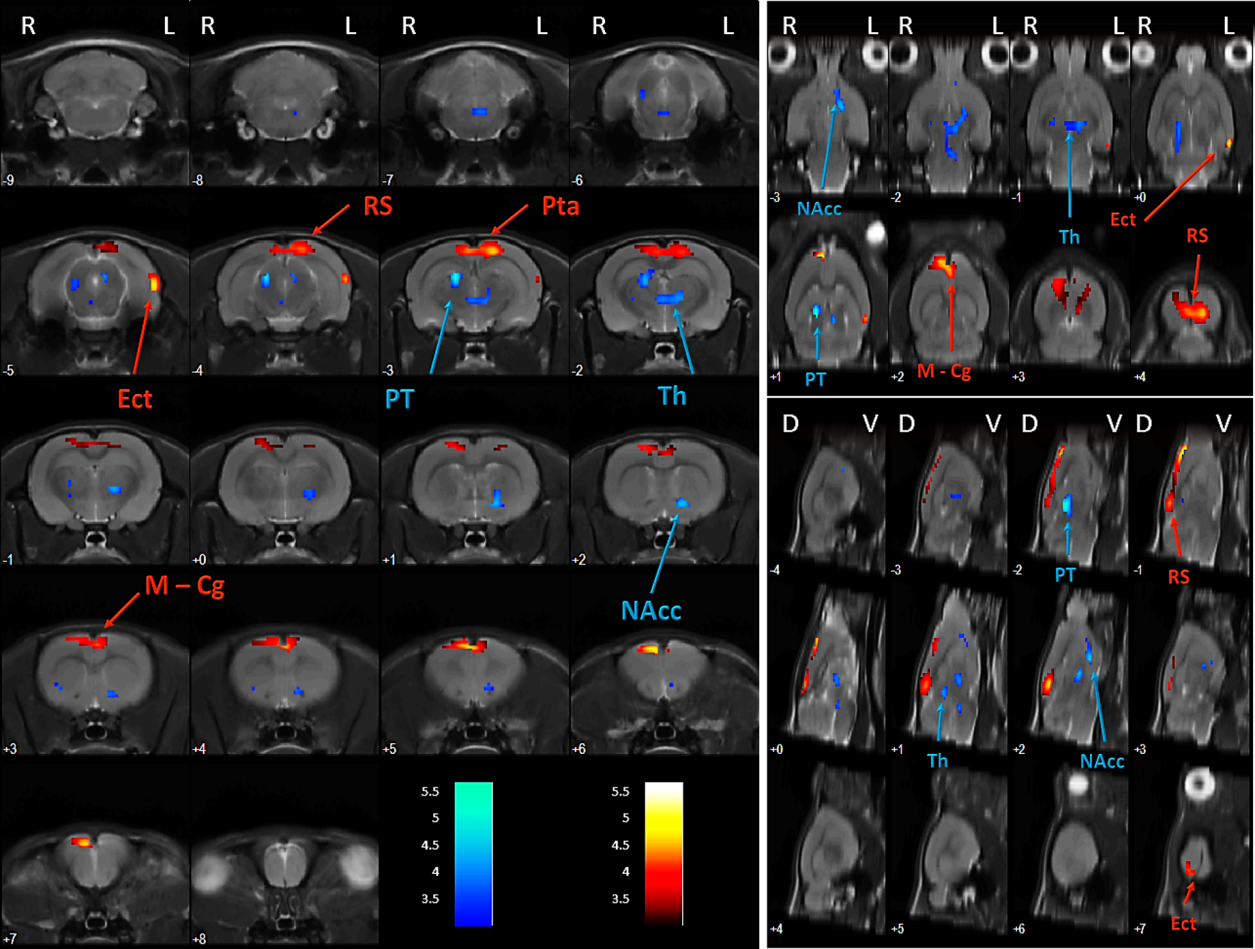
NAcc-DBS effects on brain metabolism. Axial (left), coronal (upper right) and sagittal (lower right) views of the brain. Colored PET overlays on the MR reference indicate increased FDG uptake (hot colors) or decreased FDG uptake (cold colors) 1 day after the end of the stimulation in NAcc. Left (L), right (R), dorsal (D), ventral (V).

DBS in the NAcc produced significant metabolic differences in several brain regions. In fact, voxel-by-voxel analysis revealed a decreased FDG uptake in NAcc, pretectal nucleus and thalamus (T = 5.66, p_FDR_<0.001). Moreover, an increased uptake of the radiotracer is located in a cortical cluster that comprises different portions of the cingulate, retrosplenial and parietal association cortices (T = 5.05, p_FDR_<0.001) (Fig 4, Table 1). Finally, there is also a slight hypermetabolic region in the ectorhinal–lateral entorhinal cortex (T = 4.73, p_unc_<0.001, p_FDR_ = 0.150), although it does not overcome the cluster-based correction thresholds.

**Table 1:** Changes in brain metabolic activity following 15 days of NAcc-DBS.

ROI	Hemisphere	k	T	↑/↓	p_unc._	p_FWE_	p_FDR_
NAcc—PT—Th	L & R	654	5.66	↓	< 0.001	< 0.001	< 0.001
Cg—RS—Pta	L & R	923	5.05	↑	< 0.001	< 0.001	< 0.001
Ect—LEnt	L	101	4.73	↑	< 0.001	0.253	0.150

ROI: Region of interest (Cg: cingulate cortex, Ect: ectorhinal cortex, LEnt: lateral entorhinal cortex, NAcc: nucleus accumbens, PT: pretectal nucleus, PTa: parietal association cortex, RS: retrosplenial cortex, Th: thalamus). Hemisphere: left (L) and right (R). k: cluster size, T: T Student. Glucose metabolism: increase (↑) and decrease (↓). p: p value (unc: uncorrected, FWE: family wise error, FDR: false discovery rate).

ROI analysis did not reveal statistically significant global differences in brain metabolism between sham and stimulated animals (p_SUV_>0.05), which supports the validity of WB mean intensity as a normalization method in this study. Furthermore, we found significant changes in NAcc (p<0.01), CPu (p<0.05), Th (p<0.01) and Cx (p<0.01) (Table 2).

**Table 2:** ROIs analysis results.

ROI	WB_SUV_	NAcc	CPu	Th	Cx
**Sham**	58.15 ± 8.28	1.30 ± 0.06	1.32 ± 0.05	1.20 ± 0.04	0.90 ± 0.11
**DBS**	51.38 ± 8.97	1.20 ± 0.02	1.24 ± 0.05	1.12 ± 0.01	1.08 ± 0.07
**T**	1.50	3.57**	2.91*	3.96**	3.78**

**p<0.01

*p<0.05

Data: mean ± SD, ROI: region of interest (WB: whole brain, NAcc: nucleus accumbens, CPu: caudate putamen, Th: thalamus, Cx: cortex), SUV: standardized uptake value, T: T Student

### Weight

**Fig 5:**
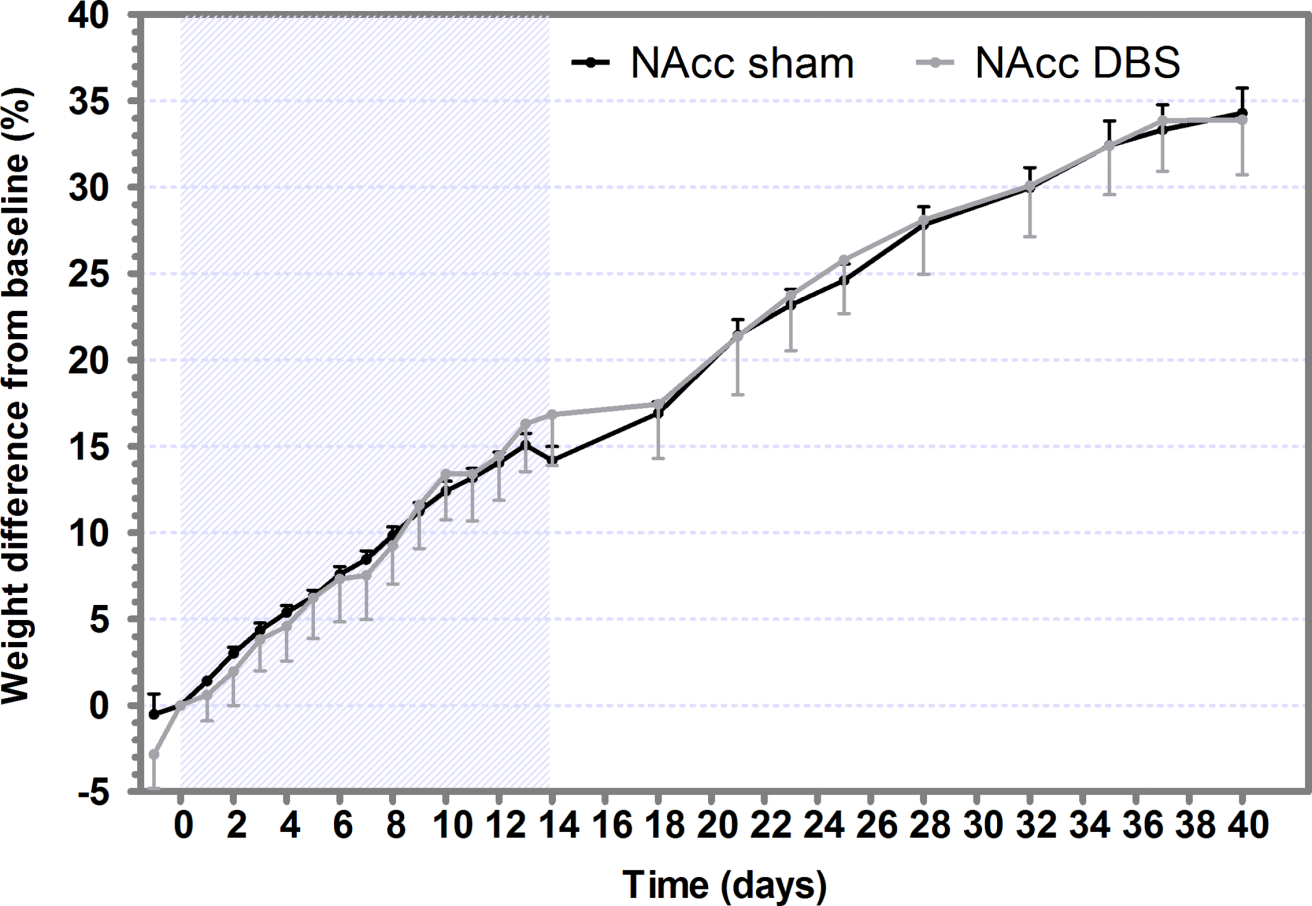
NAcc-DBS does not reduced weight gain. Weight difference (in percentage) with respect to weight recorded before DBS (baseline) in sham and DBS groups. Values are expressed as mean ± SEM. The gray-striped area indicates the DBS application period.

Neither significant differences in initial body weight were observed for the sham (382.20±19.78 g) or DBS (358.99±36.00 g) groups, nor in the weight gain between groups (Fig 5). Moreover, no statistically significant difference between slopes was found neither during the DBS treatment nor during the posterior month.

### Food and water intake

**Fig 6:**
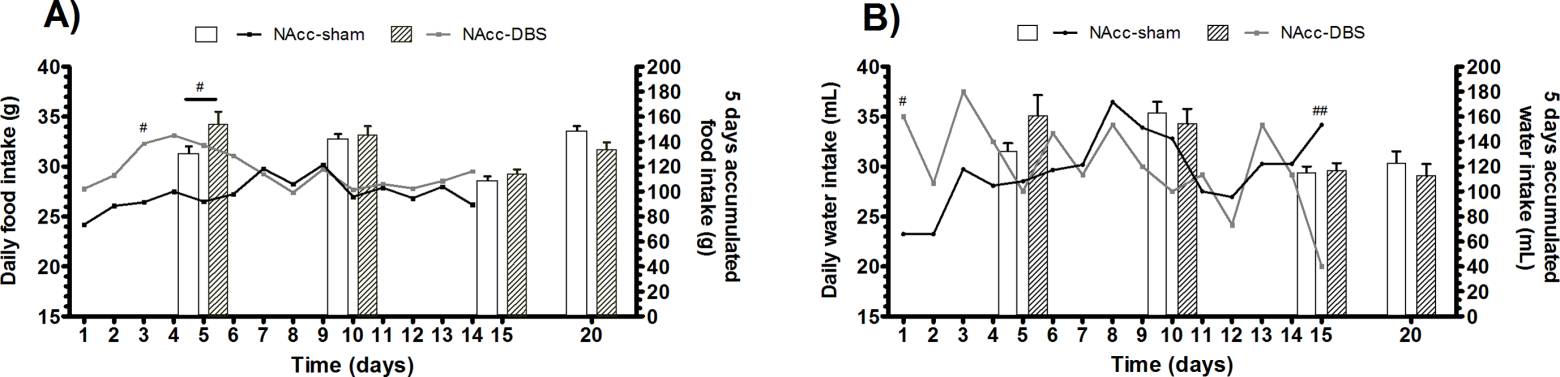
Neither food nor water intake are reduced by NAcc-DBS. Food (A) and water (B) consumption during DBS treatment in sham and DBS groups. Lines represent the mean daily intake of each group, while bars are referred to average consumption over 5 consecutive days. 5 days after stimulation are also shown for the accumulated intake (#p<0.05; ##p<0.01).

No significant differences were found in food and water intake (daily or accumulated) between groups. (Fig 6). However, both food and water intake revealed a significant effect of the time (food: F = 57.76, p<0.001; water: F = 44.02, p<0.001) and the interaction between factors (food: F = 14.54, p<0.001; water: F = 7.748, p<0.001). Moreover, post-hoc test revealed differences between groups in food intake at the beginning of the DBS treatment (daily, day 3: T = 3.01, p<0.05; accumulated, days 0 to 5: T = 3.02, p<0.05), showing reduced accumulated food intake in the sham group compared to DBS animals.

## Discussion

DBS has recently emerged as a potential therapy for treatment-resistant obesity. Thus, electrically modulating the impaired activity of brain nuclei involved in the pathophysiology of obesity, such as the NAcc, has proven to be effective in clinical studies. However, controversy regarding effectiveness can be found in the literature, and even the modulatory results for NAcc-DBS in the obese brain remain unclear. Therefore, identifying the functional consequences of NAcc-DBS could help to better understand the physiological effects of this approach and to decipher its mechanism of action.

Ours is the first study to apply small-animal FDG-PET to study brain networks undergoing 15 consecutive days of intermittent NAcc-DBS in an animal model of obesity. Thus, we showed that NAcc-DBS modulated glucose metabolism in neuronal networks related to reward and memory systems. However, we did not observe a significantly lower weight gain in animals that underwent NAcc-DBS.

### Brain metabolism

Our PET data reveal a reduction in NAcc metabolism after 15 days of intermittent stimulation (1 hour per day). This finding could be consistent with reported neural informative disruption theories [9,10]. In this sense, the action potentials produced by NAcc would be governed by the stimulation pulses, with the result that DBS would ‘capture’ NAcc activity [9].

Moreover, one day after the DBS protocol had finished, NAcc-DBS produced a modulation pattern in brain metabolism that is similar to that observed with LH-DBS [27]. In fact, NAcc-DBS decreased glucose metabolism in the caudate-putamen, an effect which has also been shown in previous reports on major depression [39,40]. These nuclei are in close communication with the cortex and mediate motor and cognitive functions [41,42]. Indeed, patients with a tendency towards obesity exhibited hyperactivation of the cortico-striato-thalamic pathway in response to a food-dependent reward feeling [43]. Similarly, food images increased glucose metabolism in the striatum of obese patients, while lowering baseline D_2_ receptor density [44]. This finding may reflect compensatory downregulation owing to the frequent transient increases in dopamine levels associated with recurrent overstimulation of the reward circuit by eating [45]. Consequently, this overstimulation might be counteracted by NAcc-DBS, as it reduced glucose metabolism in the striatum and thalamus.

NAcc-DBS also increased metabolism in the retrosplenial cortex, which plays a direct role in the consolidation of long-term memory owing to its association with the hippocampus, the parahippocampal region, and the thalamic nuclei [46–48]. Therefore, retrosplenial dysfunction could be caused by hippocampal damage and consequently contribute to the impact of hippocampal damage [49], thus supporting the idea that the thalamic nuclei depend on each other in memory and learning tasks [47,49]. Obesity has been related to defective hippocampal activity [48], which leads to cognitive deficiency in obese patients [50] and obese Zucker rats [51]. Given the strong connectivity between both structures [46], it seems reasonable that the increased metabolism observed in the cortical region might have an effect on defective hippocampal processes, thus improving the damaged memory function described in this animal model. However, behavioral experiments must be performed to corroborate these findings.

Of note, previous studies of the consequences of bilateral NAcc-DBS assessed by *in vivo* functional imaging were mainly focused on the acute effect of the NAcc-DBS (e.g. [52–55]) or applied a continuous stimulation protocol during prolonged periods [56,57], thus preventing them from being compared with the results we report here. To this end, further research should be carried out to uncover the benefits and modulatory consequences resulting from different stimulation protocols.

### Body weight and food and water intake

Given the essential role of leptin in the mesolimbic circuit, the NAcc was selected as the DBS target for the obese Zucker rat [58,59]. Consequently, leptin regulates the mesolimbic reward centers, which include the NAcc, thus promoting dopamine (DA) synthesis [59] or release [60] and inducing a food-associated reward. However, the NAcc lacks leptin receptors, and its influence is mediated by VTA and LH [20]. Therefore, obese leptin-resistant animals present impaired feelings of satiety and reward, which lead them to increase their caloric intake [61,62].

Importantly, leptin receptor is present in dopaminergic neurons of the VTA, which directly project to the NAcc and receive afferent inputs from LH neurons expressing leptin receptor [58]. In fact, the increase in DA produced by food intake in the NAcc is inhibited by leptin signals in the VTA, which also induce cessation of food intake [59]. Neto *et al*. reported lower baseline DA and serotonin levels in Zucker rats than in Wistar rats and unchanged NAcc-DA flow when leptin is intranasally administered to Zucker rats, as opposed to a clear increase in Wistar rats [63]. Furthermore, these alterations seem to be exclusive to the obese Zucker rat strain, which exhibited lower striatal DA transporter levels than their lean littermates (+/fa) [64]. These findings reinforced the idea of DAergic modulation induced by leptin in the reward system, which is directly hampered in the Zucker rat.

Given the neural disruption theories applying to the mechanism of action of DBS [9,10], stimulating the NAcc would do the following: 1) isolate this structure from the VTA and LH signals, since they promote food intake owing to the absence of leptin influence; and 2) recover normal functioning of the NAcc by directly taking charge of its activity. However, in contrast with our previous results with LH-DBS [27], NAcc-DBS did not reduce weight gain, possibly owing to the firm anorexigenic modulation of the LH and ventromedial hypothalamus by leptin [7]. In this sense, although NAcc-DBS was expected to modulate the impaired function of the reward system [65,66], it would not be able to resolve the imbalance caused by the lack of leptin signal in the hypothalamus.

In addition, van der Plasse *et al*. also reported absence of variation in average food intake when DBS was applied to the NAcc core of Wistar rats, whereas stimulation of the NAcc medial shell increased food intake [67]. Then, the fact that the stimulation could have affected both the core and the shell could explain the lack of anti-obesity effect in NAcc-DBS animals.

Importantly, the present study was based on a genetic model of obesity; in other words, a diet-induced model of obesity could show different effects. Zhang *et al*. reported that long-term DBS applied to the NAcc shell attenuated weight gain in rats with diet-induced obesity [68]. Similar results were also obtained in a mouse model of binge eating after NAcc shell DBS, although no related differences were observed after stimulating the NAcc core [22]. These results highlight the need to clarify the role of NAcc substructures before this nucleus can be considered a clinical target for DBS in obesity.

### Limitations of the study

Our study is subject to limitations. On the one hand, we cannot extrapolate the effects observed in obese Zucker rats to lean Zucker rats or other animal models of obesity. Nevertheless, our animal model is representative of a particularly resistant kind of obesity, which could potentially benefit from NAcc-DBS depending on the genetic background. We selected the DBS parameters for three main reasons: the success obtained in previous approaches using bilateral DBS [23,24] and similar stimulation protocols [22,27,34,69]; the tolerance associated with continuous DBS treatments [70,71]; and the technical difficulties in obtaining portable rat stimulators in our facilities. Nevertheless, DBS protocols which were closer to the current clinical scenario may reveal larger differences in weight gain [22,68].

## Conclusion

In conclusion, we describe an experimental approach to evaluate the neuromodulatory consequences of NAcc as a target of DBS in the treatment of obesity. Although no substantial effects in weight or intake parameters were observed, we proved that brain regions that were functionally impaired in obesity were modulated by an intermittent NAcc-DBS protocol.
